# Prenatal molecular diagnosis of β-thalassemia: report on the first two cases in Romania

**Published:** 2008-04-15

**Authors:** R Talmaci, D Coriu, L Dan, L Cherry, L Gavrila, L Barbarii, M Dogaru, F Vladareanu, R Vladareanu, G Peltecu, D Colita

**Affiliations:** * Hematology Department, University of Medicine and Pharmacy “Carol Davila”, Bucharest; ** Hematology Department, Fundeni Clinical Institute, Bucharest; *** Human Genome Department, Genetic Institute of Bucharest University, Bucharest; **** Genetic Department, National Institute of Legal Medicine, Bucharest; ***** National Institute of Transfusion Hematology, Bucharest; ****** Obstretics and Gynechology Department, “Elias” Emergency Hospital, Bucharest; ******* Obstretics and Gynechology Department, “Filantropia” Clinical Hospital, Bucharest

**Keywords:** Prenatal diagnosis, beta-thalassemia, amniocentesis, CVS, DNA analysis, genotype, phenotype, DGGE, ARMS-PCR, PCR-RFLP

## Abstract

Thalassaemia major is a classical example of a disease that can be prevented by prenatal diagnosis. In Romania there are currently 300 patients with thalassaemia major under the management of specialized institutions. Prenatal diagnoses of thalassemia have offered a new dimension to the prevention of this disease, but in order to implement prenatal diagnosis, knowledge of mutations and of their incidence is essential.

Molecular testing using Denaturing Gradient Gel Electrophoresis (DGGE) scanning and direct mutation detection with Amplificaton Refractory Mutation System-PCR (ARMS-PCR) and Restriction endonuclease Analysis of PCR fragments (PCR-RFLP) was performed by using amplified DNA from amniotic cells samples, while mutations in the parents were determined in advance.

Using our experience in molecular diagnosis, we were able to perform the first prenatal diagnosis for two young couples at risk for thalassaemia major. Foetal samplings were collected by amniocentesis and chorionic villus sampling in the second trimester of the pregnancies. Maternal contamination of the foetal DNA was ruled out by STR genotyping. The prenatal diagnosis revealed affected foetuses with homozygous status of β-thalassemia major. The IVSI-110 (G-A)/IVS II-745 (C-G) genotype in the first case foetus and cd 8 (-AA)/cd 8 (-AA) in the second case foetus were reported.

The results of this study point to a successful future prenatal diagnosis of beta-thalassaemia in Romania, using a rapid and accurate molecular method. Together with the implementation of proper preventive health measures and the education of parents regarding their carrier status, we are hoping that this method will be used as the common application approach to decrease the incidence of thalassaemia major.

## Introduction

The thalassaemia syndromes are a heterogeneous group of genetic disorders in which the production of normal haemoglobin is partly or completely suppressed because of defective synthesis of one or more globin chain [**1**]. The wide distribution and extended prevalence of β-thalassaemia major in the Middle and Far East make it the most common of all inherited human lethal conditions. Over 5% of the world’s population are healthy carriers of a haemoglobin disorder, and worldwide about 60,000 children with major thalassaemia are born annually [**2**]. It is transmitted as a Mendelian recessive; hence heterozygous individuals are asymptomatic but can be reliably diagnosed by blood tests. When two heterozygous individuals marry, the risk of giving birth to an affected child is one in four for each pregnancy. Untreated, such patients usually die of anaemia between 2 and 6 years of age. Effective treatment consists of regular blood transfusions and intensive administration of iron-chelating agent desferrioxamine by subcutaneous infusions [**3**]. Early prenatal diagnosis of a foetus at risk of Beta thalassaemia major is essential for pregnant women and their partners to have sufficient time to think through the available options. Usually the heterozygous state for these conditions does not give rise to clinical symptoms but the homozygous state frequently results in severe, debilitating lifelong-illness.

In Romania there are about 300 patients with thalassaemia major while this disease represents an important health problem due to its high rate of early childhood mortality. So far, prenatal molecular diagnosis was not available in our country. Knowledge of a population mutation spectrum is important in order to implement a molecular diagnosis and to build a strategy for carrier screening in the framework of a national prevention program for high-risk couples [**4**, **5**]. Molecular analysis revealed a striking heterogeneity of β-thalassaemia genes mutations, about 200 different mutation types being reported so far [**6**]. Population studies indicate that 20 β-thalassaemia alleles account for > 80% of mutations. Due to geographical clustering phenomenon, each population is characterized by few common mutations. More than 8 different mutations are reported in the Romanian population [**7**].

Here, we report on two cases of high-risk couples solved by molecular diagnosis methods. To the best of our knowledge, this is the first time when molecular testing was performed in Romania in order to investigate a foetus at risk for severe β-thalassaemia disease. Foetal DNA isolated from non-cultured amniotic fluid cells and CVS during the second trimester of pregnancies were analysed for β-thalassaemia mutations. First, mutation scanning DGGE method of entire β-globin gene was performed. Secondly, results were verified with ARMS-PCR and PCR-RFLP methods.

## Materials and methods

Blood samples from the pregnant women and their husbands, diagnosed as carriers by Hb electrophoresis, were brought to our laboratory for β-thalassaemia mutation analysis. The genetic molecular testing for parents was performed in advance. The amniotic fluid sample was obtained by transabdominal amniocentesis, and the CVS were obtained by biopsy of chorionic villi, both in the second trimester of pregnancies. Participants in this study gave their informed consent.

**Blood sample preparation**

1 ml of each EDTA blood sample was processed to isolate DNA using QIAamp DNA Blood Mini Kit (QIAGEN-USA). The DNA was quantified by means of a spectrophotometer (Perkin Elmer-USA) obtaining 30μg/µl.

**Foetal sample preparation**

Amniotic fluid sample (about 5 ml) was centrifuged at 2500g for 15 minutes and cell pellet was twice washed with TE before further processing. For CVS, the villi were viewed under a microscope and carefully separated from any tissue that was of doubtful (maternal) origin, placed in 1 ml of TE buffer in sterile 1.5ml Eppendorf tubes and washed twice prior to further processing.

Foetal DNA was isolated from the cell pellet or CVS using QIAamp DNA Mini Kit (QIAGEN). The foetal DNA samples were then quantified by means of a spectrophotometer (Perkin Elmer) obtaining up to 10μg/μl of DNA.

**Assessment of maternal DNA contamination**

It was performed in the Genetics Department - National Institute of Legal Medicine, Bucharest. The foetal DNA samples were compared with the mother’s DNA obtained from a reference blood sample using a set of 16 STR loci - IdentifilerTM kit (*Applied Biosystems*). The PCR reactions were performed according to the manufacturer protocols in a GeneAmp PCR System 9700 (*Applied Biosystems*). PCR products were detected by capillary electrophoresis on an ABI 3100 Avant Genetic Analyzer (*Applied Biosystems*) using the allelic ladders provided by the PCR kit supplier. Typing results were analysed by means of the GeneMapper® 3.2 software (*Applied Biosystems*). The allele designation for the STR loci followed the recommendations of the International Society of Forensics Genetics [**8**].

*Quality control for STR typing:* Proficiency testing certificates from the GEDNAP -ISFG working group.

**Mutation analysis**

Once the common mutations in a population have been previously identified [**5**], molecular techniques like DGGE, ARMS-PCR, PCR-RFLP were applied for fast and accurate detection of these mutations. In our protocol, parental mutations were characterized prior to foetal DNA analysis and a foetal sample was subsequently assayed alongside the parental samples as controls.

**Fig. 1:**
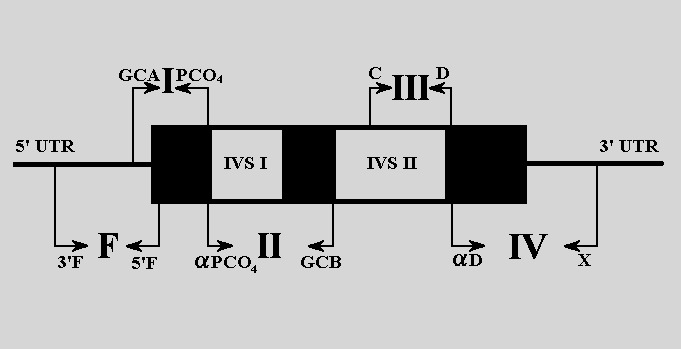
PCR amplification scheme for β-globin gene. F, I, II, III, IV - gene fragments analyzed by PCR; exons - black areas; introns - white areas; PCR primers sites - arrows.

Five β-globin gene fragments were amplified using different sets of primers including whole structural and un-translated regions of β-globin gene, described by Kanavakis et al [**9**]. (**Fig. 1**1).

**• PCR Amplification**

Amplification of genomic DNA was performed with HotStarTaq Master Mix Kit Qiagen in GeneAmp PCR System 2400 thermal-cycler (*Perkin Elmer*):

-2.5 units HotStarTaq DNA Polymerase;

-1x PCR buffer;

-200 mM of each dNTP;

-0.1-0.5 mM of each primer;

-1μl DNA template.

PCR conditions were: incubation at 95ºC for 15 min, followed by 35 cycles of: 95ºC – 1 minute, 60ºC – 1 minute, 72ºC – 1.30 minutes and final extension at 72ºC for 8 minutes.

**• Denaturing gradient gel electrophoresis (DGGE)**

10 μl of PCR products were analysed using the DGGE protocol previously described [**9**]. The 6% polyacrilamide gels with a linearly increasing gradient from 35% to 75% denaturant (100% denaturant - 7M urea/40% formamide) were run in a DGGE-2000 system (*C.B.S. Scientific*). The electrophoresis was performed at 50V for 16 hours. After electrophoresis, the gel was stained with ethidium bromide and examined under UV trans-illumination.

**• Amplification refractory mutation system (ARMS)**

To identify the mutant alleles, DNA was amplified for a IVS I-110 (G-A) mutation and for cd 8 (-AA) sets of ARMS primers (*Applied Biosystems*), as previously reported by Old [10], using a GeneAmp PCR System 2400 (*Perkin Elmer*). Each PCR reaction mix contained 1μl genomic DNA, 10mM Tris, pH 8.3, 50mM KCl, 1.5mM MgCl2, 0.01% (w/v) gelatin, 0.2mM each of dATP, dCTP, dGTP, dTTP, as well as Taq polimerase (1 unit). One pair of ARMS primers and a control primer pair (10pmol of each) were also included in a total reaction volume of 25μl. PCR conditions were: 25 cycles of 95ºC – 1 minute, 65ºC – 1 minute, 72ºC – 1 minute and a final extention at 72ºC for 3 minutes. PCR products were ascertained by agarose gel electrophoresis 2%.

**• Restriction enzyme analysis or PCR-RFLP**

The IVS II-745 (C-G) β-thalassaemia mutation was characterized by taking advantage of the gain of additional ***Rsa I*** cutting site. The β-globin gene fragment III (Fig. 1) was amplified and PCR products were digested with the help of *Rsa I* restriction enzyme digestion. Each restriction reaction mix containing 25μl PCR amplification product, 3μl enzyme *Rsa I* and 3μl restriction buffer was incubated over night at 37ºC. Products sizing was performed by agarose gel electrophoresis 2%.

## Results

**For the first case**

**Fig. 2:**
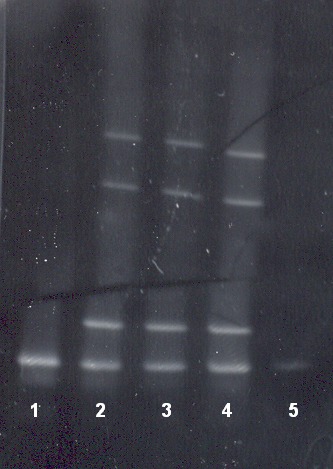
DGGE patterns of fragment I. 1-mother DNA (negative), 2-father DNA (positive control), 3-grandfather DNA (positive control), 4-foetal DNA positive for IVS II-745 mutation, 5-grandmother DNA (negative).

**Fig. 3:**
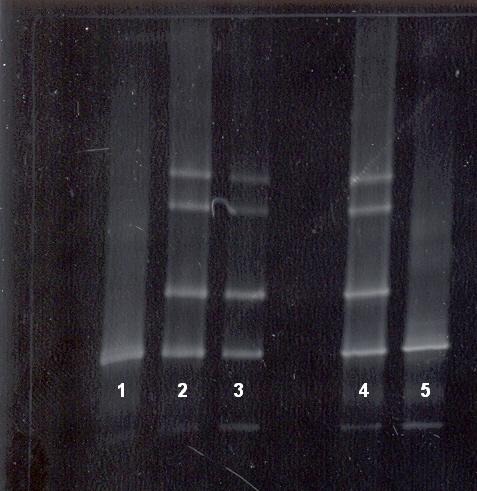
DGGE patterns of fragment II. 1-grandmother DNA (negative), 2 and 3- foetal DNA positive for IVS I-110 (G-A) mutation, 4-mother DNA (positive control), 5-negative control DNA.

The β-globin gene, virtually separated in five fragments, was analysed (**Fig. 1**). Three regions (fragment F, I and II) of the β-globin gene were amplified and analysed by DGGE (**Fig. 2**, **3**). The fragment III was amplified and analysed for IVS II-745 (C-G) mutation by means of restriction analysis PCR-RFLP (**Fig. 5**).

Prior to diagnosis of foetal sample, the parents’ mutations had to be characterized. Based upon the haematological phenotype of each individual, appropriate techniques were selected from amongst five PCR-based methods described in the methodology in order to genotype each parental, and subsequently each foetal sample.

In the case of a heterozygous state four bands were seen in the gel. Among them, two bands situated upward represent hetero-duplexes, while the remaining two bands, situated downward represent homo-duplexes. In the homozygous state (normal or mutant) only one band is present at the specific site (**Fig. 2**).

The DGGE pattern was compared to a particular pathological mutation from the control samples. In an accurate manner, DGGE cannot be used to define the presence of mutation in a sample, even when unknown samples were analysed next to familiar controls, as sometimes different mutations show very similar (undistinguishable) DGGE patterns.

Before foetal DNA analysis, we performed the molecular analysis of the couple’s entire family. In fragment I, the DGGE patterns revealed the presence of IVS II-745 (C-G) mutation in foetal DNA having the father’s and grandfather’s DNA as positive controls (**Fig. 2**).

DGGE patterns in fragment II identified the IVS I-110 (G-A) β-thalassaemia mutation in foetal DNA having the mother’s DNA as positive control (**Fig. 3**).

**Fig. 4:**
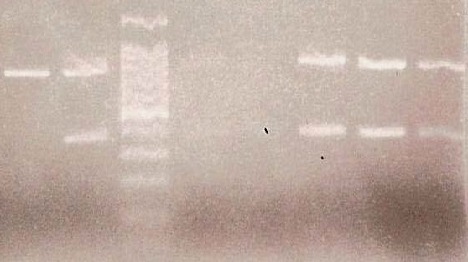
PCR specific amplification of IVS I-110 (G-A) allele. 1-negative control DNA, 2-positive control DNA (IVS I-110/N), 3 and 4-fetal DNA (positive), 5-mother DNA (positive), MW-molecular weight marker

In order to confirm our results obtained by DGGE we have applied two other molecular methods. Thus the DGGE positive samples of IVS I-110 (G-A) mutation were analysed by means of the ARMS-PCR method (**Fig. 4**). All samples under investigation were analysed simultaneously, alongside positive and negative controls for the IVS I-110 (G-A) mutation (**Fig. 4**).

**Fig. 5:**
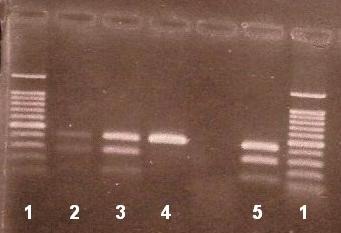
PCR-RFLP pattern of IVS II-745 (C-G) mutation. Restriction enzyme digestion of amplified fragment III by *Rsa* I. 1- MW, 2 and 3-fetal DNA positive for IVS II-745 (C-G) mutation, 4-negative control DNA, 5-father DNA (positive control).

The DGGE positive samples of IVS II-745 (C-G) mutation were verified by means of the RFLP-PCR method. Results from restriction enzyme analysis were assessed in comparison with positive and negative controls for the mutation IVS II-745 (C-G), analysed simultaneously (**Fig. 5**).

Molecular diagnosis of parents and grandparents revealed the presence of β-thalassaemia mutations. (**Table 1**)

**Fig. 6:**
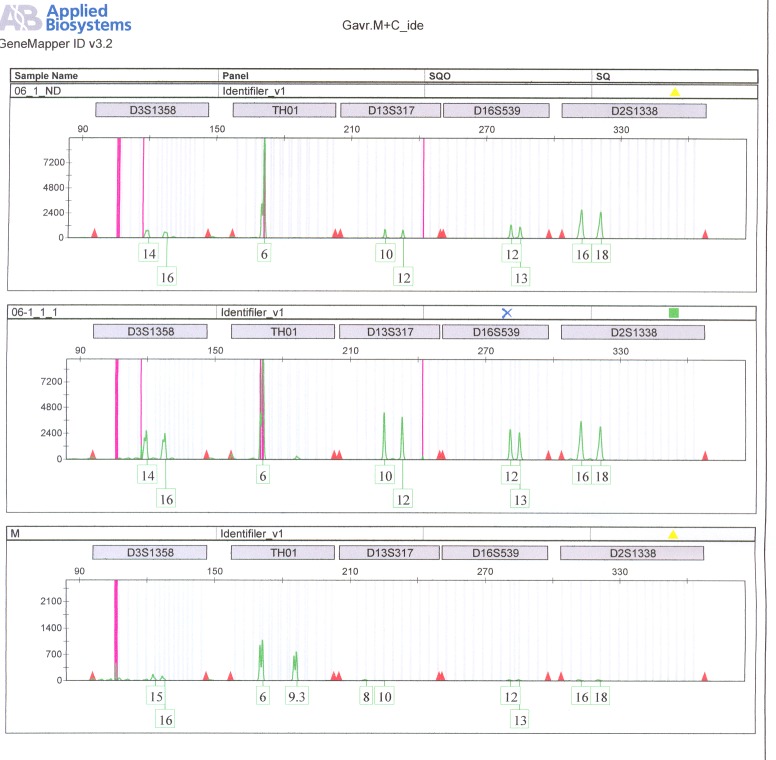
DNA profiles obtained by STR typing. Profiles of the amniotic fluid sample (06-1 ND and 06-111) are different from the maternal blood sample (M) appearing to be free of any maternal contamination.

DNA profiles obtained by STR typing of the maternal blood sample and of the amniotic fluid sample are presented in **Figure 6**. The analysis revealed a profile for the foetal DNA sample, free of any maternal contaminants.

**For the second case**

**Fig. 7:**
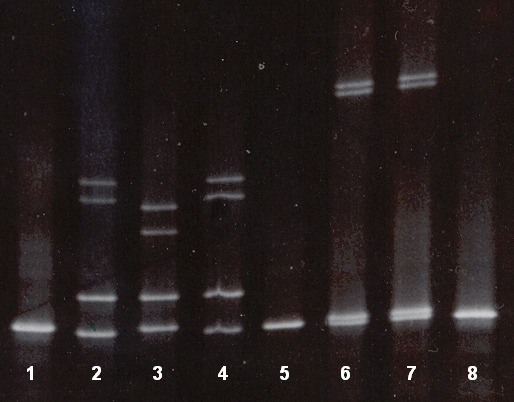
DGGE patterns of fetal DNA and parents’ DNA in fragment I. 1-Normal DNA (negative control); 2-foetal DNA from amniotic fluid sample (shows the comtamination with maternal DNA), 3–heterozygous cd 2 polymorphism (positive control), 4–mother DNA (cd 8 (-AA) mutation + cd 2 polymorphism in trans, 5–foetal DNA from CVS (homozygous cd 8 (-AA) mutation; 6–father DNA (heterozygous cd 8 (-AA) mutation; 7–heterozygous cd 8 (-AA) mutation (positive control); 8–homozygous cd 8 (-AA) mutation (positive control).

The fragment I of the β-globin gene was positive in both parents in the second case of prenatal diagnosis. This fragment I of the β-globin gene was amplified and analysed by DGGE. The mother and father both had heterozygous state for cd 8 (-AA) β-thalassaemia mutation, in addition the mother was positive for cd 2 polymorphism (*in trans*) also. The foetus had inherited both cd 8 (-AA) alleles from his mother and father in homozygous state (**Fig. 7**). Compared to the parents’ mutations, the foetal DNA from CVS revealed a cd 8/cd 8 DGGE pattern. The foetal DNA extracted from the amniotic fluid was excluded from analysis, having been contaminated with maternal DNA (line 2, **Fig. 7**).

**Fig. 8:**
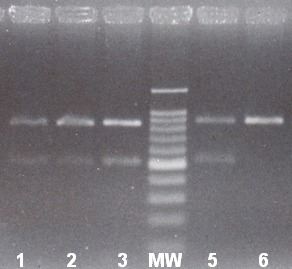
PCR specific amplification of cd 8 (-AA) allele. 1–fetal DNA from CVS positive for cd 8 (-AA) mutation; 2–mother DNA (positive control); 3–father DNA (positive control), 4-molecular weight marker; 5-foetal DNA from CVS (positive); 6–negative control DNA.

Using the ARMS-PCR method, the mutant β-thalassaemia allele cd 8 (-AA) was identified. The method was applied for confirmation of mutations detected by DGGE method. All samples under investigation were analysed simultaneously, alongside positive and negative controls for the cd 8 (-AA) mutation (**Fig. 8**).

Molecular diagnosis of the second case family revealed the presence of cd 8 (-AA) β-thalassaemia mutation. (**Table 2**)

**Fig. 9:**
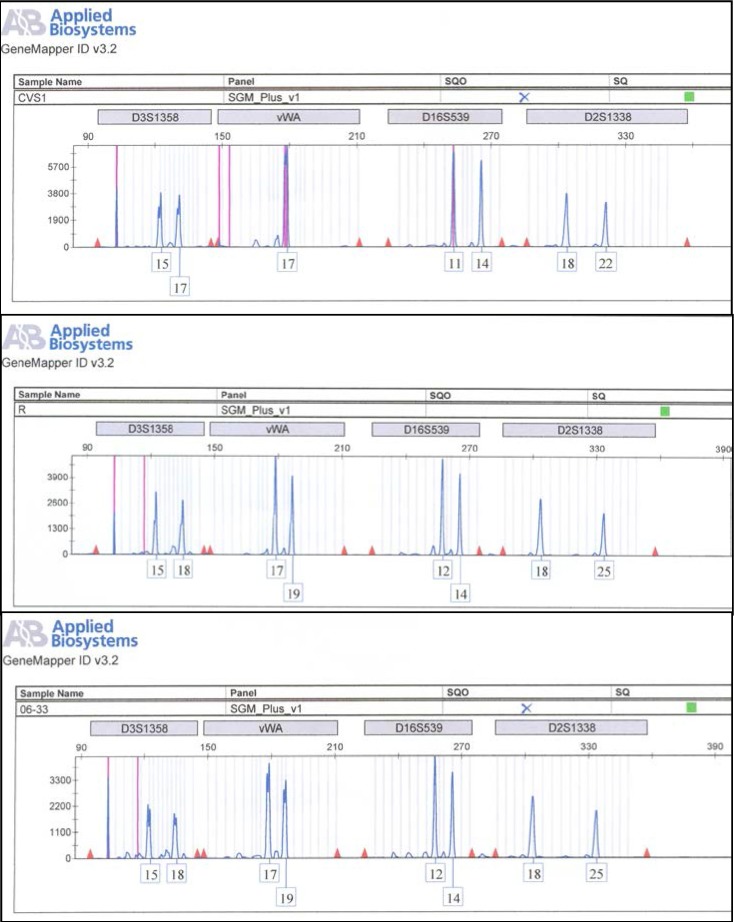
DNA profiles obtained by STR typing. The profile of CVS sample (CVS1) is different from the amniotic fluid sample (R) and from the maternal blood sample (06-33) appearing to be free of maternal contamination. The profile of amniotic fluid sample matches the mother’s profile showing a contamination.

In this case, DNA profiles obtained by STR typing revealed a contamination of amniotic fluid sample DNA with maternal DNA (**Fig. 9**), as the foetus DNA fully matched the mother’s profile. The CVS foetal DNA samples appeared free of any maternal contaminants and only these samples were further considered in prenatal diagnosis.

## Discussion

DGGE was the primary method used for screening the β-globin gene in the heterozygous parents to localize pathological mutations. Most β-gene mutations are associated with distinct electrophoretic patterns. This first step allowed us to select the appropriate methods to specifically characterize the mutations. 

In the first case, molecular analysis for foetal DNA samples identified a homozygous foetus with IVS I-110/IVS II-745 genotype (β+/β+ phenotype). Both IVS I-110 (G-A) and IVS II-745 (C-G) β-thalassaemia mutations cause a reduction (β+) of β-globin production. β-thalassaemia mutation IVS I-110 (G-A) gives the formation of a new splicing site resulting in an abnormal spliced mARN. On the other hand, mutation IVS II-745 (C-G) also creates a new splicing site, which is preferentially used. In this case, no normal β-globin protein is produced.

In the second case, molecular analysis of foetal DNA samples identified also homozygous genotype cd 8/cd8 with β0/β0 phenotype. β-thalassaemia mutation cd 8 (-AA) is a frame shift mutation that blocks the mARN synthesis. In this case (homozygous state) no β-globin chains are produced.

In some cases (such as our second case), amniotic fluid samples are contaminated with maternal DNA. Such maternal contamination can be ruled out by the analysis of hyper-variable DNA polymorphisms or STR analysis. In our second case just from CVS sample results was considered.

We have selected a couple of methods for a higher efficiency in order to offer a rapid and accurate prenatal DNA diagnosis service for molecular genetic disease.

Our first results in this approach were confirmed by the DNA analysis performed in The National Thalassaemia Centre from Athens, Greece.

## Tables

**Table 1:** Results of molecular diagnosis of the first case family

	PHENOTYPE	GENOTYPE
FOETUS	Homozytoge for IVS I-110 (G-A) and IVS II-745 (C-G) mutations	.β+/β+
MOTHER	heterozygote for IVS I-110 (G-A) mutation	.β+/βA
FATHER	heterozygote for mutation IVS II-745 (C-G)	.β+/βA
GRANDFATHER	heterozygote for IVS II-745 (C-G) mutation	.β+/βA
GRANDMOTHER	Normal	.βA /βA

**Table 2:** Results of molecular diagnosis of the second case family

	PHENOTYPE	GENOTYPE
FOETUS	Homozygote for cd 8 (-AA)/cd 8 (-AA) mutation	.β0/β0
MOTHER	heterozygote for cd 8 (-AA) mutation and cd 2 polymorphism	.β0/βA
FATHER	heterozygote for cd 8 (-AA) mutation	.β0/βA

## Conclusions

The complex molecular strategy applied for prenatal diagnosis in the reported cases was a successful one. The foetus β-thalassaemia profiles were fully characterized leading to a recommendation for an induced abortion.

To the best of our knowledge, these are the first cases of β-thalassaemia prenatal diagnosis using DNA testing methods reported in Romania. Our department can now perform β-thalassaemia genetic testing within a few working days following the receipt of the samples. On this basis, we are currently considering the possibility to develop a national prevention program for β-thalassaemia and other haematological genetic disorders.

Additional studies are required to fully assess the β-globin gene polymorphism in the Romanian population and to estimate the population’s level of β-thalassaemia mutations. Such studies are currently undergoing in our department.

## Acknowledgements 

The authors wish to gratefully acknowledge Ms. Sinopoulou Klio and his medical team from The National Thalassaemia Centre, Laikon General Hospital in Athens, Greece for helping us confirm the prenatal diagnosis. We express our thanks to Mrs. Ramona Cherciu for the English language proofreading.

*This work was supported by National Research Grants CEEX 48/2005, 49/2005, 74/2006; CNCSIS 201; PN II 41-045 and European FP6-ITHANET*.
